# Cervical vestibular evoked myogenic potential (cVEMP) findings in adults with sensorineural hearing loss (SNHL): comparisons between 500 Hz tone burst and narrowband CE-Chirp stimuli

**DOI:** 10.1038/s41598-023-48810-1

**Published:** 2023-12-21

**Authors:** Mohd Normani Zakaria, Rosdan Salim, Nor Haniza Abdul Wahat, Mohd Khairi Md Daud, Wan Najibah Wan Mohamad

**Affiliations:** 1 Audiology Programme, School of Health Sciences, Universiti Sains Malaysia, 16150 Kubang Kerian, Kelantan Malaysia; 2 Department of Otorhinolaryngology, School of Medical Sciences, Universiti Sains Malaysia, 16150 Kubang Kerian, Kelantan Malaysia; 3 Centre for Rehabilitation and Special Needs, Faculty of Health Sciences, Universiti Kebangsaan Malaysia, 50300 Kuala Lumpur, Malaysia

**Keywords:** Medical research, Neurology

## Abstract

There has been a growing interest in studying the usefulness of chirp stimuli in recording cervical vestibular evoked myogenic potential (cVEMP) waveforms. Nevertheless, the study outcomes are debatable and require verification. In view of this, the aim of the present study was to compare cVEMP results when elicited by 500 Hz tone burst and narrowband (NB) CE-Chirp stimuli in adults with sensorineural hearing loss (SNHL). Fifty adults with bilateral SNHL (aged 20–65 years) underwent the cVEMP testing based on the established protocol. The 500 Hz tone burst and NB CE-Chirp (centred at 500 Hz) stimuli were presented to each ear at an intensity level of 120.5 dB peSPL. P1 latency, N1 latency, and P1–N1 amplitude values were analysed accordingly. The NB CE-Chirp stimulus produced significantly shorter P1 and N1 latencies (*p* < 0.001) with large effect sizes (*d* > 0.80). In contrast, both stimuli elicited cVEMP responses with P1–N1 amplitude values that were not statistically different from one another (*p* = 0.157, *d* = 0.15). Additionally, age and hearing level were found to be significantly correlated (*r* = 0.56, *p* < 0.001), as were age and cVEMP amplitude for each stimulus (*p* < 0.001). To conclude, since both stimuli were presented at an equivalent intensity level (in dB peSPL), the shorter P1 and N1 latencies of cVEMP produced by the NB CE-Chirp stimulus (centred at 500 Hz) were unlikely due to enhanced saccular stimulation. Another more sensible reason is the temporal adjustment of the chirp stimulus.

## Introduction

It has been demonstrated that the otolith organs (i.e., saccule and utricle) are responsive to sounds^1–3^. The cervical vestibular evoked myogenic potential (cVEMP) is an electrophysiological procedure that is able to assess the function of the saccule, the inferior vestibular nerve, and the respective central pathway^3^. As such, it is useful to identify saccular dysfunction and impaired inferior vestibular nerve in various vestibular disorders^2,3^.

Since the inner ear and otolithic organs are in close proximity, cVEMP responses have been studied in those with and without hearing loss. In fact, it was also of interest to ascertain whether the prominent peaks of cVEMP (i.e., P1 and N1) could be of cochlear origin. In 1994, Colebatch and his colleagues were the first to report that robust cVEMP waveforms could still be recorded from patients with bilateral severe sensorineural hearing loss (SNHL)^4^. This implies that the saccular afferents had no “relationship” with the status of cochlea. Other subsequent studies corroborated this finding. In particular, Ozeki et al. observed the presence of cVEMP waveforms in all patients with bilateral profound hearing loss^5^. Likewise, this result suggests that the cVEMP responses were not likely of cochlear origin. In a study by Wu and Young, cVEMP results were compared between those with unilateral idiopathic sudden hearing loss and healthy control participants^6^. In the tested group, the amplitudes and latencies of cVEMP were found to be not statistically different between the affected and normal ears. These results also did not differ significantly from those of the control group. The authors then concluded that the cVEMP responses were not related to the hearing status^6^. Takegoshi and Murofushi recorded cVEMP and auditory brainstem response (ABR) waveforms from one ear, while a white noise was given ipsilaterally and contralaterally to the tested ear^7^. They then found that when the noise was presented at 75 dB nHL, only a minor reduction in cVEMP amplitudes was observed (5% reduction), whereas ABR amplitudes were significantly reduced (23% reduction). Similar to the previous reports, this finding suggests that the peaks of cVEMP were independent of cochlear afferents. In a study by Wu and Young, 91.7% of patients with acute low-tone SNHL were found to have normal cVEMP waveforms^8^. The high response rate (i.e., the percentage of cVEMP responses) in those with SNHL would again support the notion that cVEMP results were unaffected by the cochlear status. Contradictorily, considerable percentages of abnormal cVEMP results have been reported in participants with SNHL due to cochlear damage^9–11^. In a study by Sazgar et al.^9^ cVEMP responses to click stimuli were found to be absent in 44.7% of patients with unilateral or bilateral high-frequency SNHL (while only 15.6% of normal adults revealed no identifiable cVEMP peaks). In accordance with this, Akin and her colleagues recorded cVEMPs from normal adults and noise-exposed subjects with SNHL^10^. They then found that 33% of SNHL subjects had abnormal cVEMP results and a significant association was found between the severity of hearing loss and cVEMP outcomes. However, the authors concluded that the abnormal cVEMP waveforms were due to damaged sacculocollic pathway (where cochlea was also affected due to the noise exposure)^10^. In a study by Xu et al.^11^ while healthy participants revealed a 100% response rate, only 44.4% of ears in patients with profound SNHL produced cVEMP responses. These findings reveal that those with hearing loss had a high incidence of damaged otolithic organs. Collectively, it appears that the relationship between hearing status and saccular function in patients with SNHL is unclear. Until further evidence emerges, the contribution of cochlear to the cVEMP generation is currently not well justified.

Given its diagnostic performance, having the cVEMP testing can be beneficial in clinical settings. However, due to its myogenic nature, measurable cVEMP responses can only be obtained when the respective sternocleidomastoid (SCM) muscle is adequately contracted (i.e., by asking the tested patient to turn his/her head away from the stimulated ear)^3^. In this regard, the patient might not feel comfortable if the testing takes a long time. As such, it is advantageous to have the most optimal stimulus so that robust cVEMP waveforms can be attained quicker. It is also clinically important to have robust waveforms to support the validity and reliability of the assessment tool and enhance testers’ confidence in results interpretation^12^. In line with this, many studies have demonstrated low frequency tone bursts to be effective in eliciting cVEMP responses^13–15^. Relative to other conventional stimuli, such as clicks, the low frequency tone bursts have been consistently shown to produce cVEMP responses with larger amplitudes. Owing to this, these stimuli have been recommended to be used for clinical applications^3^.

More recently, there has been a growing interest to study the usefulness of chirp stimuli in the cVEMP testing^16–26^. Of note, the chirp stimuli were specifically designed to enhance auditory brainstem response (ABR) amplitudes by compensating for the cochlear travelling wave delay^27,28^. Most of the studies found the P1–N1 amplitude of cVEMP to be statistically larger when elicited by the narrowband (NB) CE-Chirp stimulus (centred at 500 Hz), relative to the conventional 500 Hz tone burst^18,21–24^. More notably, the P1 and N1 latencies were found to be significantly shorter for the chirp stimulus^17,18,20–26^. Given the positive results of the chirp stimulus, it can be a viable alternative stimulus to be used in clinical settings. However, more credible evidence is required to support this viewpoint.

Among healthy participants, it has been consistently demonstrated that the largest cVEMP amplitudes are typically produced by the 500 Hz tone burst^15,29,30^. Smaller amplitudes are observed for tone bursts below or above this frequency. However, it is worth noting that this response pattern may not be applicable to all age groups. Unlike young adults, studies have shown that in older adults, larger cVEMP amplitudes are seen at higher frequencies^31–33^. In a study by Akin et al., cVEMP responses to 500 Hz tone burst were recorded from young (aged 22–31 years, mean = 24.3 ± 2.5 years) and older (aged 61–86 years, mean = 70.5 ± 5.8 years) adults^31^. As revealed, the cVEMP amplitudes were found to be statistically smaller for the older adults across different electromyography (EMG) target levels. In line with this, Piker et al. observed that while most of young adults (aged 18–39 years) revealed largest cVEMP amplitudes when elicited by either 500 Hz or 750 Hz, older adults (aged ≥ 60 years) revealed maximum amplitudes when evoked by either 750 Hz or 1000 Hz^32^. Jha et al.^33^ demonstrated that for middle-aged and older adults, an upward shift in the frequency tuning of cVEMP was noted, in which 750 Hz and 1000 Hz tone bursts yielded higher response rates and larger amplitudes. Taken together, among other factors, the frequency of the stimulus should be taken into consideration when recording cVEMP from participants of different ages.

Many possible reasons have been proposed to explain why the chirp stimuli could produce more robust cVEMP waveforms (i.e., shorter latencies and larger amplitudes)^18,21–26^, but they remain to be proven. Wang et al. pioneered a study to compare the performance of NB CE-Chirp (centred at 500 Hz), 500 tone burst, and click stimuli in recording cVEMP responses from healthy adults^18^. They postulated that the shorter P1 and N1 latencies for the chirp stimulus were due to enhanced activation of the saccule that would occur as a result of increased movement of the endolymph (as the chirp stimulus would effectively stimulate the basilar membrane of the cochlea). The shorter cVEMP latencies elicited by chirp stimuli have been consistently demonstrated in the majority of studies since then^17,20–26^. Most of the studies have also revealed that significantly larger cVEMP amplitudes are evoked by the chirp stimuli^18–24^. Several reasons have been proposed to explain this finding. Nevertheless, the stimulus intensity factor has not been highlighted as the potential reason. It is worth stating that the majority of studies have compared the cVEMP amplitudes elicited by chirp and 500 Hz tone burst stimuli presented at the same dB nHL^17–26^. In this regard, even though the two stimuli have an equivalent intensity level (as measured in dB nHL), it is possible for the saccule to be exposed to different sound pressure levels, resulting in different cVEMP amplitudes. As such, to have a more accurate comparison, the intensity level for both stimuli should be presented at the same dB peak equivalent (pe) SPL (i.e., dB peSPL).

Despite the fact that some other explanations have been put forth, it is interesting to note many studies did not contradict the viewpoint proposed by Wang et al. (that the shorter cVEMP latencies for the chirp stimulus could be due to enhanced activation of the saccule, as a result of increased movement of the endolymph). This postulation, nevertheless, can be debatable for several reasons. From one perspective, this postulation asserts that the saccule would be stimulated by the chirp stimulus just as effectively as the basilar membrane is (which is rather insensible given that the chirp stimulus was mathematically designed to enhance auditory neural synchrony based on the physiological aspect of the cochlea). Furthermore, if this postulation were true, the enhanced stimulation of the saccule would not occur (and longer cVEMP latencies would be obtained) if the cochlea is damaged (as the increased auditory neural synchrony would not take place). In view of this, it can be advantageous to investigate whether the P1 and N1 latencies (elicited by the chirp stimulus) would be altered in individuals with impaired cochlear. Therefore, the aim of the present study was to compare the cVEMP results between NB CE-Chirp (centred at 500 Hz) and 500 Hz tone burst stimuli in adults with SNHL. The outcomes of this study would contribute to a better understanding of the “relationship” between cochlea and saccule, as well as provide additional evidence to verify the “superiority” of the chirp stimulus in recording cVEMPs. Additionally, this study also aimed to determine the correlations between age, hearing loss, and cVEMP results among the participants.

## Materials and methods

### Participants

In the present cross-sectional study, all methods were carried out in accordance with relevant guidelines and regulations. Fifty eligible adults with bilateral SNHL (aged 20–65 years) were enrolled, and they were selected among the patients of the Otorhinolaryngology Clinic, University Hospital. The respective informed consent was obtained from all participants prior to the data collection. All of them denied having any vestibular symptoms during the history taking, and their scores were within the normal limit on the Malay version of vertigo symptom scale (MVVSS)^34^. The hearing diagnosis was made based on established clinical assessments. In particular, by means of pure tone audiometry (PTA), their air conduction (AC) and bone conduction (BC) thresholds exceeded 20 dB HL with no significant air-bone gaps (ABGs) bilaterally. Despite having normal middle ear function (by means of tympanometry), they also had abnormal results in the distortion product otoacoustic emissions (DPOAE) test (indicating dysfunction of outer hair cells of the cochlea) bilaterally.

### cVEMP procedure

All participants received detailed instructions prior to the testing. The Eclipse EP25 device (Interacoustics, Assens, Denmark) was used to record cVEMP waveforms. For the placement of electrodes, a non-inverting electrode was placed at the midline of the left and right SCM muscles, an inverting electrode was placed on the upper sternum, and a ground electrode was placed on the participant’s forehead. The impedance of all electrodes was kept below 5 kΩ during the testing.

The cVEMP testing was carried out in a soundproof room, with the participant seated comfortably on a provided chair. To contract the SCM muscle, he/she was asked to rotate his/her head to the side opposite the test ear. To achieve sufficient SCM muscle activation, the visual feedback method was used (by ensuring that the rectified electromyography (EMG) level was at least 50 µV). Two stimuli were employed, i.e., 500 Hz tone burst (6 ms duration: 2 ms rise/fall time and 2 ms plateau time) and NB CE-Chirp (360–720 Hz, centred at 500 Hz, 9 ms duration). They were delivered to each ear (monaurally) using insert earphones (ER-3A, Etymotic Research, Illinois, USA), at a repetition rate of 5.1/s and at an intensity level of 120.5 dB peSPL (i.e., 95 dB nHL and 97 dB nHL for NB CE-Chirp and 500 Hz tone burst stimuli, respectively). For each trial, the cVEMP responses were averaged after 200 sweeps and filtered at 10–1000 Hz. The time window between − 20 and 80 ms was used to inspect the waveforms. Each trial was repeated twice in order to achieve good waveform reproducibility.

### Data analysis

The cVEMP data were expressed in percentage, mean, and standard deviation (SD). The comparison of response rates between the two stimuli was carried out using the McNemar test. Since the data were found to be normally distributed (*p* > 0.05 by the Shapiro–Wilk test), the paired t-test was conducted to compare the cVEMP results between left and right ears. This analysis was again employed to compare the cVEMP data between the two stimuli. A *p* value of less than 0.05 was considered statistically significant. To support the *p* value approach, the Cohen effect size (*d*) was applied to determine the magnitude of difference when comparing the cVEMP findings between the two stimuli. In particular, a small effect is defined if *d* = 0.20, a medium effect if *d* = 0.50, and a large effect if *d* = 0.80^35^. To measure the correlations between the variables, the Pearson correlation analysis was conducted. The correlation coefficient (*r*) of < 0.20 indicates very weak correlation, 0.20–0.39 suggests weak correlation, 0.40–0.59 denotes moderate correlation, 0.60–0.79 indicates strong correlation and > 0.80 implies very strong correlation^36^. The JASP statistical software (version 0.17.1, University of Amsterdam, Netherlands) was used for the data analysis.

### Ethical standard

This study was approved by Human Ethics Committee of Universiti Sains Malaysia (USM/JEPeM/21100693).

## Results

**Figure 1:**
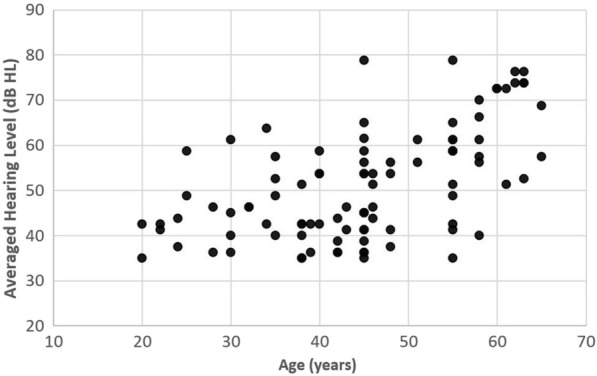
A scatter plot showing the relationship between age and hearing level of participants.

The participants enrolled in the present study had a mean age of 45.5 years (SD = 12.0 years). All of them were Malay, and 58.0% were women. They were found to have SNHL bilaterally (due to impaired cochlea), and the averaged hearing loss at four frequencies (i.e., 500, 1000, 2000, and 4000 Hz) ranged from 35.6 dB HL to 78.8 dB HL (mean = 53.9 ± 12.1 dB HL). It is worth mentioning when the participants were divided into three different age groups, i.e., younger (20–39 years), middle-aged (40–59 years) and older (≥ 60 years), there were 15, 27, and 8 participants in each group, respectively. Because the sample size was not equal across the groups (and the older group had the most insufficient sample size), the correlation analysis (instead of ANOVA) was chosen to determine the relationship between age and hearing level. As revealed, a moderate positive correlation was found between the two variables (*r* = 0.56,* p* < 0.001). The scatter plot for this analysis is shown in Fig. 1.

**Figure 2:**
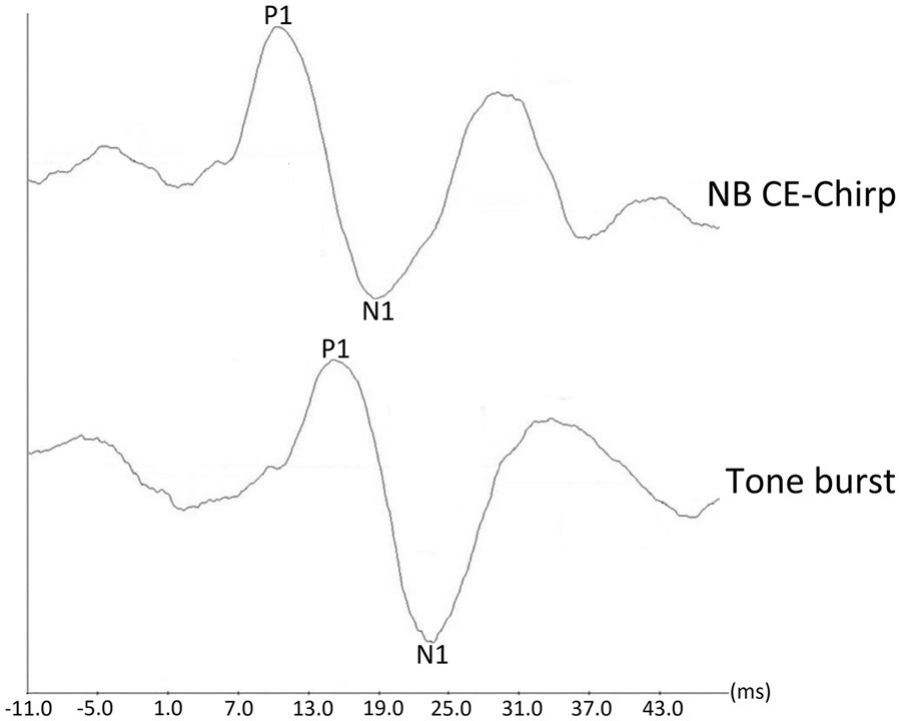
Waveforms of cervical vestibular evoked myogenic potential (cVEMP) elicited by 500 Hz tone burst and narrow band (NB) CE-Chirp (centred at 500 Hz) stimuli from a representative participant.

Figure 2 shows cVEMP waveforms elicited by both stimuli from a representative participant. The response rates of cVEMP were 94.0% and 96.0% for 500 Hz tone burst and NB CE-Chirp stimuli, respectively. The response rates of both stimuli did not differ significantly (*χ*^*2*^(1, *n* = 50) = 0.00, *p* = 1.000). Since the cVEMP results were found to be not statistically different between left and right ears (*p* > 0.05) (Table 1), the data were then pooled (i.e., *n* = 94 ears for the 500 Hz tone burst and *n* = 96 ears for the NB CE-Chirp).

**Table 1:** Mean, standard deviation (SD), and paired t-test results when cervical vestibular evoked myogenic potential (cVEMP) results are compared between left and right ears for each stimulus.

cVEMP parameter	Ear	500 Hz tone burst (mean ± SD)	NB CE-Chirp (mean ± SD)
P1 latency (ms)	Left	16.77 ± 2.60	10.23 ± 2.58
	Right	16.16 ± 2.06	10.55 ± 1.74
	*p* value	0.138	0.471
N1 latency (ms)	Left	24.61 ± 2.87	18.46 ± 2.77
	Right	24.93 ± 2.19	19.10 ± 2.02
	*p* value	0.538	0.226
P1–N1 amplitude (µV)	Left	80.75 ± 32.76	81.12 ± 34.61
	Right	74.99 ± 30.74	78.74 ± 32.76
	*p* value	0.341	0.757

Table 2 shows the cVEMP results when both stimuli were compared. As revealed, relative to the 500 Hz tone burst stimulus, the NB CE-Chirp stimulus produced significantly shorter P1 and N1 latencies (*p* < 0.001). This finding was supported by large effect sizes (*d* > 0.80), indicating that the differences were indeed substantial. Conversely, the P1–N1 amplitude values were found to be comparable between the two stimuli (*t*(91) = 1.427, *p* = 0.157), with a small effect size (*d* = 0.15).

**Table 2:** Mean, standard deviation (SD), and the respective statistical outcomes (i.e., *p* value and Cohen effect size, *d*) when cervical vestibular evoked myogenic potential (cVEMP) results are compared between the 500 Hz tone burst stimulus (*n* = 94 ears) and the narrowband (NB) CE-Chirp stimulus (centred at 500 Hz) (*n* = 96 ears).

cVEMP parameter	500 Hz tone burst (mean ± SD)	NB CE-Chirp (mean ± SD)	*p* value	Effect size (*d*)
P1 latency (ms)	16.47 ± 2.25	10.45 ± 2.24	< 0.001*	3.25
N1 latency (ms)	24.80 ± 2.49	18.77 ± 2.41	< 0.001*	3.26
P1–N1 amplitude (µV)	77.90 ± 31.73	78.96 ± 33.10	0.157	0.15

*Significant at *p* < 0.05.

Additionally, no significant relationship was found between age and cVEMP latencies for both stimuli (*r* = 0.02–0.15,* p* > 0.05). Conversely, a moderate negative correlation was noted between age and P1–N1 amplitude for each stimulus (*r* = − 0.52, *p* < 0.001 and r = − 0.54, *p* < 0.001 for 500 Hz tone burst and NB CE-Chirp, respectively). Meanwhile, for both stimuli, no significant association was found between hearing level and P1–N1 amplitude (*p* > 0.05,* r* = − 0.18 and *r* = − 0.16 for 500 Hz tone burst and NB CE-Chirp, respectively) or P1 and N1 latencies (*r* = 0.04–0.19, *p* > 0.05).

## Discussion

In the present study, the response rates of both 500 Hz tone burst and NB CE-Chirp (centred at 500 Hz) stimuli in recording cVEMP waveforms were high. This finding was rather anticipated, as the cVEMP responses were thought to be not mediated by the cochlea and could still be present in cases of profound SNHL^2–8^. As reported by the previous studies on adults with SNHL, the response rates of cVEMP ranged from 44.4 to 100.0%^6,8,9,11,37,38^. The high response rates in those with SNHL obtained in the present study are in accordance with the previous reports^6,8^. In particular, Wu and Young found that cVEMP responses were present in all ears with SNHL (i.e., a 100.0% response rate, *n* = 20)^6^. Likewise, in another study, cVEMP waveforms were also observed in 100.0% of patients with SNHL (*n* = 12)^8^. In the present study, the response rate for each stimulus was close to 100.0% (94.0% and 96.0% for 500 Hz tone burst and NB CE-Chirp stimuli, respectively). Compared to the previous studies^6,8^, the present study recruited a larger sample size (*n* = 50), and this can be a potential reason as to why a 100.0% response was not achieved. In contrast, lower response rates of cVEMP among adults with SNHL were reported by other studies^9,11,37,38^. In a study by Hong et al., measurable cVEMP responses were observed in 84.6% of patients with sudden idiopathic SNHL (without vertigo)^37^. Meanwhile, in a study by Tseng and Young involving individuals with noise induced hearing loss, the response rate of cVEMP was 63.3%^38^. Sazgar et al. reported a lower response rate of cVEMP (i.e., 55.3%) while studying those with high-frequency SNHL^9^. Methodological differences between the studies could explain the disparities in study findings. In the present study, 500 Hz tone burst and chirp stimuli were used to elicit cVEMP responses, while the studies by Sazgar et al.^9^ and Hong et al.^37^ employed click stimuli for evoking cVEMP waveforms. As reported elsewhere, relative to the click stimuli, tone bursts have been consistently found to produce cVEMP responses with larger amplitudes and higher response rates^3,14,15,18,23^. Unlike the present study, Tseng and Young utilized a bone vibrator to record cVEMP responses, and this might contribute to different study findings^38^. A much lower response rate of cVEMP was reported by Xu and colleagues^11^. Particularly, they found that the cVEMP waveforms were present in only 44.4% of ears in those with profound SNHL (and all normal participants produced cVEMP responses). Several likely reasons might explain the differences in the study findings. First, the number of patients with SNHL in the study by Xu et al. was lower than that of the present study (i.e., *n* = 29 vs. *n* = 50). Second, in the study by Xu et al., it is possible that those with SNHL also had vestibular problems (leading to more abnormal cVEMP results), as 74.1% of them revealed abnormal results in the caloric test. In this regard, further well-controlled studies (with larger sample sizes) are warranted to ascertain the relationship between the cochlea and saccular response.

The P1 latency, N1 latency, and P1–N1 amplitude values obtained in the present study are in the range reported by previous studies that also employed low frequency tone bursts when assessing normal and SNHL participants^6,17,20–23,39^. Since this is the first study to employ the NB CE-Chirp stimulus for recording cVEMP responses from adults with SNHL, it is therefore challenging to compare the obtained data with other studies. Nevertheless, the cVEMP findings provided by the chirp stimulus are indeed in accordance with those of previous studies involving healthy adults^22,23,25^.

Recall that Wang et al.^18^ postulated that the shorter cVEMP latencies elicited by the chirp stimulus could be due to enhanced activation of the saccule, as a result of increased movement of the endolymph. It is to be noted that the ability of the chirp stimulus to stimulate the basilar membrane effectively (leading to enhanced neural synchronization) has been well demonstrated^27,28^. However, it is not known whether the enhanced stimulation of the basilar membrane would also increase the activation of the saccule in response to the chirp stimulus (given that the cochlea and the saccule are different anatomically and physiologically). In the present study, the P1 and N1 latencies elicited by the NB CE-Chirp stimulus were indeed significantly shorter than those of the 500 Hz tone burst. A similar pattern was also observed in the studies that compared both stimuli among healthy adults^17,18,20–26^. In view of this, it appears that the cVEMP waveforms with shorter latencies could still be obtained, even when the cochlea was impaired (i.e., the enhanced activation of the basilar membrane did not occur). Therefore, the viewpoint proposed by Wang et al. was insufficient to explain the occurrence of the shorter cVEMP latencies elicited by the chirp stimulus^18^. Furthermore, even if the enhanced stimulation was supposed to occur in the saccule (in response to the chirp stimulus), there is no physiological explanation as to why shorter latencies were observed (as well as why the latency difference between the two stimuli was rather large, i.e., around 6 ms). Another viewpoint is that since the tone burst has a long duration, vestibular neurons may show double or triple firing (in response to a single tone burst)^22,23,40^. In this regard, the latencies of cVEMP waveforms (elicited by the tone burst) may be prolonged due to the delay in neural firing^40^. This opinion is worthy of consideration if a comparison is made between the long-duration tone burst and short-duration click stimuli. That is, because of its short duration, significantly shorter cVEMP latencies have been consistently produced by the click stimulus^13–15^. However, the “neural firing delay” postulation seems inapplicable to explain why shorter cVEMP latencies were noted for the chirp stimulus. This is because there is no evidence that the chirp stimulus (which has a longer duration than the tone burst) would excite the respective neurons faster than the tone burst. Until further evidence becomes available, the most likely reason for the shorter cVEMP latencies for the chirp stimulus is rather “technical”, i.e., the commercially available chirp stimulus was placed earlier than the respective tone burst^16,23,41^. It is worth mentioning that the NB CE-Chirp stimuli were designed by Elberling and Don, and the onset of these stimuli was adjusted to be earlier than the zero point (0 ms) (resulting in waveforms with shorter latencies)^28^.

Consistent with the findings of previous studies^25,26^, the present study also demonstrated that the P1–N1 amplitude of cVEMP was not statistically different between the two stimuli. Nevertheless, other studies found that the NB CE-Chirp (centred at 500 Hz) produced significantly larger P1–N1 amplitude values^18,21–23^. The use of intensity levels reported in dB nHL can be one of the potential reasons to explain this. This intensity scale is less appropriate as it reflects cochlear sensitivity, and the application of dB peak equivalent (pe) SPL is recommended^3^. Moreover, at 0 dB nHL, the NB CE-Chirp (centred at 500 Hz) and 500 Hz tone burst stimuli produce intensity levels of 25.5 dB peSPL and 23.5 dB peSPL, respectively^42^. This demonstrates that at a similar dB nHL, the chirp stimulus would deliver a comparatively higher sound pressure level to the saccule. Even though the intensity difference is small (about 2 dB), it appears possible to have larger P1–N1 amplitude values due to this. In the present study, both stimuli were presented at the same intensity level (i.e., 120.5 dB peSPL) in an effort to stimulate the saccule equally (and comparable amplitude values were indeed obtained).

Additionally, in the present study, a significant association was found between the age and hearing level of the participants. This finding is sensible given that hearing loss is typically more prevalent and severe in older adults due to the aging effect^43–45^. The present study also found that older adults tended to have lower cVEMP amplitudes relative to younger and middle-aged adults. This finding is in line with those of the previous studies on the influence of stimulus frequency on cVEMP amplitude in adults of different ages^30–33^. That is, given that the “best” frequency to elicit cVEMP waveforms in older adults was higher (i.e., 750 Hz or 1000 Hz), lower cVEMP amplitudes were seen when the 500 Hz tone burst was used as the stimulus^30–33^. In line with the findings of the previous studies, no significant relationship was noted between age and cVEMP latencies^30,31^. Meanwhile, it was found that there was no significant correlation between the hearing levels of participants and cVEMP results. This lack of association again supports the notion that the cVEMP results were not influenced by the cochlear status^4–8^.

The present study had several limitations. First, the number of subjects for young, middle-aged, and older adults was not evenly distributed. Perhaps better outcomes could be obtained if the sample size for each age group is made comparable. Second, in line with most of the previous studies, the 500 Hz tone burst used in the present study was 6 ms long, while the NB CE-Chirp stimulus was 9 ms long. In this regard, for a better comparison, it can be interesting to observe the cVEMP results if the duration of the 500 Hz tone burst is also set at 9 ms (similar to the chirp stimulus), and this is subject to further research.

## Conclusion

Since the relationship between cVEMP and cochlea remains unclear, the main aim of the present study was to provide evidence that the shorter cVEMP latencies produced by the NB CE-Chirp stimulus (centred at 500 Hz) were unlikely due to the status of cochlea. As revealed, relative to the 500 Hz tone burst, significantly shorter P1 and N1 latencies of cVEMP were elicited by the chirp stimulus in adults with SNHL (due to cochlear damage). In view of this, the postulation that shorter chirp-evoked cVEMP latencies were due to the enhanced stimulation of the saccule (as a result of increased movement of the endolymph) was not in favour. The other reason, such as the temporal adjustment of the chirp stimulus (i.e., it is placed earlier than the respective tone burst), is more justifiable. The cVEMP amplitude was also found to be comparable between the two stimuli, implying that the chirp stimulus was not necessarily superior to the conventional tone burst. Additionally, the age of participants was found to be positively correlated with their hearing level and negatively correlated with the cVEMP amplitude.

## Data Availability

The datasets used and/or analysed during the current study available from the corresponding author on reasonable request.
